# Preserved central cholinergic functioning to transcranial magnetic stimulation in *de novo* patients with celiac disease

**DOI:** 10.1371/journal.pone.0261373

**Published:** 2021-12-16

**Authors:** Giuseppe Lanza, Francesco Fisicaro, Carmela Cinzia D’Agate, Raffaele Ferri, Mariagiovanna Cantone, Luca Falzone, Giovanni Pennisi, Rita Bella, Marios Hadjivassiliou, Manuela Pennisi

**Affiliations:** 1 Department of Surgery and Medical-Surgical Specialties, University of Catania, Catania, Italy; 2 Clinical Neurophysiology Research Unit, Oasi Research Institute-IRCCS, Troina, Italy; 3 Department of Biomedical and Biotechnological Sciences, University of Catania, Catania, Italy; 4 Gastroenterology and Endoscopy Unit, University Hospital Policlinico “G. Rodolico-San Marco”, Catania, Italy; 5 Department of Neurology, Sant’Elia Hospital, ASP Caltanissetta, Caltanissetta, Italy; 6 Epidemiology and Biostatistics Unit, Instituto Nazionale Tumori-IRCCS “Fondazione G. Pascale, Napoli, Italy; 7 Department of Medical and Surgical Sciences and Advanced Technologies, University of Catania, Catania, Italy; 8 Academic Department of Neurosciences, Sheffield Teaching Hospitals NHS Foundation Trust, Royal Hallamshire Hospital, Sheffield, United Kingdom; University of Ottawa, CANADA

## Abstract

**Background:**

Celiac disease (CD) is now viewed as a systemic disease with multifaceted clinical manifestations. Among the extra-intestinal features, neurological and neuropsychiatric symptoms are still a diagnostic challenge, since they can precede or follow the diagnosis of CD. In particular, it is well known that some adults with CD may complain of cognitive symptoms, that improve when the gluten-free diet (GFD) is started, although they may re-appear after incidental gluten intake. Among the neurophysiological techniques, motor evoked potentials (MEPs) to transcranial magnetic stimulation (TMS) can non-invasively probe *in vivo* the excitation state of cortical areas and cortico-spinal conductivity, being also able to unveil preclinical impairment in several neurological and psychiatric disorders, as well as in some systemic diseases affecting the central nervous system (CNS), such as CD. We previously demonstrated an intracortical disinhibition and hyperfacilitation of MEP responses to TMS in newly diagnosed patients. However, no data are available on the central cholinergic functioning indexed by specific TMS measures, such as the short-latency afferent inhibition (SAI), which might represent the neurophysiological correlate of cognitive changes in CD patients, also at the preclinical level.

**Methods:**

Cognitive and depressive symptoms were screened by means of the Montreal Cognitive Assessment (MoCA) and the 17-item Hamilton Depression Rating Scale (HDRS), respectively, in 15 consecutive *de novo* CD patients and 15 healthy controls. All patients were on normal diet at the time of the enrolment. Brain computed tomography (CT) was performed in all patients. SAI, recorded at two interstimulus intervals (2 and 8 ms), was assessed as the percentage amplitude ratio between the conditioned and the unconditioned MEP response. Resting motor threshold, MEP amplitude and latency, and central motor conduction time were also measured.

**Results:**

The two groups were comparable for age, sex, anthropometric features, and educational level. Brain CT ruled out intracranial calcifications and clear radiological abnormalities in all patients. Scores at MoCA and HDRS were significantly worse in patients than in controls. The comparison of TMS data between the two groups revealed no statistically significant difference for all measures, including SAI at both interstimulus intervals.

**Conclusions:**

Central cholinergic functioning explored by the SAI of the motor cortex resulted to be not affected in these *de novo* CD patients compared to age-matched healthy controls. Although the statistically significant difference in MoCA, an overt cognitive impairment was not clinically evident in CD patients. Coherently, to date, no study based on TMS or other diagnostic techniques has shown any involvement of the central acetylcholine or the cholinergic fibers within the CNS in CD. This finding might add support to the vascular inflammation hypothesis underlying the so-called “gluten encephalopathy”, which seems to be due to an aetiology different from that of the cholinergic dysfunction. Longitudinal studies correlating clinical, TMS, and neuroimaging data, both before and after GFD, are needed.

## Introduction

Within the wide spectrum of gluten-related disorders [1], it is now established that the classical celiac disease (CD) represents only the tip of the “CD iceberg” [2], since for each typical patient 5-to-6 additional subjects exhibit non-typical phenotypes [3]. As such, CD is currently viewed as a systemic disease with multifaceted clinical manifestations.

Among the extra-intestinal features, neurological and neuropsychiatric symptoms are still a diagnostic challenge, given that they can precede or follow the diagnosis of CD [1, 4–6]. In a recent prospective study of newly diagnosed CD patients [7], neurological deficits were common and a significant volume decrease in some cerebral regions was observed in those with positive transglutaminase-6 antibodies. It has been also demonstrated that most of confirmed CD subjects referred for neurological consultation may already show changes at brain magnetic resonance imaging (MRI) [8]. Therefore, a reliable diagnostic tool, allowing an early detection, progression monitoring, and assessment of complications underlying the disease is needed.

Among the neurophysiological techniques, motor evoked potentials (MEPs) to transcranial magnetic stimulation (TMS) can non-invasively probe *in vivo* the excitation state of cortical motor areas [9, 10], the conduction along the cortico-spinal tract [11], and the functional connectivity across hemispheres [12]. Clinically introduced as a diagnostic tool to study the central motor pathway, today TMS goes well beyond the mere assessment of the cortico-spinal tract, being also employed to map motor and cognitive functions, to explore neural networks, and to modulate brain activity with a potential therapeutic aim [13]. Although not always clinically evident, the involvement of motor areas in cognitive disorders has been shown by clinical, neuropathological, and neuroimaging studies. Namely, changes in the motor areas may be secondary to direct structural alterations caused by the disease process itself but, more often, they are the consequence of indirect remodeling mechanisms [14]. In this context, increasing evidence supports the hypothesis that the phenomena of brain plasticity are involved in different types of dementia, related to functional and structural components, each entailing a number of cellular mechanisms operating at different time scales, synaptic loci, and developmental phases, within an extremely complex framework [9, 15].

More recently, TMS-derived parameters have allowed to support the concept of a wider cortical motor network, with the output also influenced by non-primary motor areas, including the ventral and dorsal premotor cortex, supplementary motor area, and cingulate cortex [16]. In particular, it is known that the cingulate cortex, together with the dorsolateral prefrontal cortex, is crucial for cognition and mood regulation [17]. Accordingly, TMS is able to unveil preclinical motor impairment in several neurological and psychiatric disorders, as well as in some systemic diseases affecting the central nervous system (CNS), also providing clues on prognosis [18] and treatment [19].

Finally, the so-called “pharmaco-TMS” may distinctively explore transmission pathways within the CNS, such as those mediated by gamma-aminobutyric acid (GABA), glutamate, acetylcholine, and monoamines, through the administration of selective drug agonists or antagonists [20–24]. Indeed, the application of a single dose of a CNS active drug with a defined mode of action is useful to explore and characterize the pharmaco-physiological properties of TMS measures of motor cortical and cortico-spinal excitability in humans. With this approach, it has been demonstrated that different TMS measures reflect not only axon excitability but also specific inhibitory or excitatory synaptic excitation state of distinct neuronal elements within the CNS [21, 25]. As such, pharmaco-TMS has opened an exciting window into human cortical physiology and pathophysiology.

To date, however, no data are available on the central cholinergic functioning in CD indexed by specific TMS measures, such as the short-latency afferent inhibition (SAI). Briefly, by coupling peripheral nerve stimulation with TMS of the contralateral primary motor cortex (M1), it is possible to recruit specific neuronal circuits within the human brain. In particular, it has been demonstrated that the median nerve stimulation at the wrist is able to suppress the MEP, evoked by TMS 18–21 ms later, in relaxed hand muscles. This effect, called SAI, is produced by some inhibitory interactions within the brain [26]. Namely, SAI has been related to the central cholinergic activity, given that in normal subjects it can be reduced or abolished by the muscarinic antagonist scopolamine, it is abnormal in cholinergic forms of dementia and, in these patients, it can be restored by cholinergic drug intake [27]. Therefore, SAI represents a non-invasive way of probing the functioning of central cholinergic cortical circuits [28]. Indeed, SAI abnormalities have been found in Alzheimer’s disease (AD) [29], and the fact that these changes have been observed also in early AD stages [29] and even in amnestic mild cognitive impairment (MCI) [30] has recently raised relevant diagnostic and therapeutic implications. Nevertheless, recent pharmaco-TMS evidence on the effects of lorazepam (a GABA-A agonist) and baclofen (a GABA-B agonist) on both SAI and long-latency afferent inhibition (LAI) reveals also a GABAergic modulation of SAI, thus advancing our understanding of the electrophysiological mechanisms and neurochemistry underlying afferent inhibition [31].

In this context, it is well known that some adults with CD may complain of cognitive symptoms, usually in terms of a “brain fog”, that improve when the gluten-free-diet (GFD) is started, although they may re-appear after incidental gluten intake [32, 33]. Difficulties in attention and concentration, lapses in episodic memory and word-retrieval, decreased mental acuity, and episodes of disorientation or “confusion” are also commonly reported complaints [34]. In some severely affected cases, an overt dementia can develop [34–37]. Nevertheless, most of the previous studies usually included heterogeneous cohorts, at different disease phases, or without controls.

In this cross-sectional study, we aimed to evaluate SAI to TMS in *de novo* CD patients compared to age-matched healthy controls. We hypothesized that these subjects might exhibit changes in SAI, even preclinically.

## Materials and methods

### Subjects and assessment

Fifteen consecutive *de novo* patients with CD (13 women; mean age ± standard deviation (SD): 34.07 ± 12.03 years), diagnosed according to the European Society for Pediatric Gastroenterology Hepatology and Nutrition guidelines [38], were enrolled from the Regional Center for Celiac Disease of the Azienda Ospedaliero-Universitaria “*Policlinico G*. *Rodolico-San Marco*” of Catania (Italy). Fifteen age-matched healthy individuals (13 women; 33.80 ± 9.29 years) served as a control group. All patients were right-handed and on normal diet at the time of the enrolment. Their disease duration prior to the diagnosis was 3.64 ± 1.78 years (mean ± SD).

Exclusion criteria were: age < 18 years; any CNS (i.e., Parkinson’s disease, stroke, dementia, traumatic brain injury, multiple sclerosis, epilepsy, etc.) or psychiatric disease (major depressive disorder, bipolar disorders, schizophrenia, obsessive–compulsive disorder, etc.); chronic, acute, or severe medical conditions (i.e., heart failure, coronary heart disease, liver or kidney failure, etc.); illicit drug abuse or alcohol dependency; intake of drugs influencing mood or M1 excitability (i.e., antidepressants, benzodiazepines, mood stabilizers, antipsychotics); pacemaker, pregnancy, or any other condition precluding MEP recording, according to the latest guidelines of the International Federation of Clinical Neurophysiology (IFCN) on TMS safety [39].

The clinical and demographic assessment included: age, sex, educational level, handedness, general and neurological exams, comorbidities. A screening test of the global cognitive status by means of the Montreal Cognitive Assessment (MoCA), adjusted for age and educational level for each individual [40], and an estimation of depressive symptoms through the 17-item Hamilton Depression Rating Scale (HDRS) [41] were performed by an operator (F.F.) blind to the participant status as patient or control. A brain computed tomography (CT) was also acquired in all patients with a helical 64-slice General Electric scanner (2.5 mm slice thickness) to detect intracranial calcifications (that can be found in CD) and to exclude clear neuroradiological lesions.

The Ethics Committee of the Azienda Ospedaliero-Universitaria “*Policlinico G*. *Rodolico-San Marco*” of Catania (Italy) approved the study (approval code: Prot. n.103/694). Informed consent was signed by each individual prior to the participation in accordance with the Declaration of Helsinki in 1964 and subsequent amendments. Every procedure was carried out in a dedicated laboratory by experienced operators.

### TMS procedure

TMS was carried out by means of a high-power, Magstim 200 stimulator (Magstim Co., Whitland, Dyfed, UK). A 70 mm figure-of-eight coil was positioned with the handle pointing positioned backwards and laterally, at an angle of 45° to the sagittal plane, and on the optimum site of stimulation within the M1 of the left hemisphere at the best position of the scalp to evoke MEPs in the First Dorsal Interosseous (FDI) muscle of the contralateral side, according to the Edinburgh Handedness Inventory (EHI) [42]. A biphasic pulse system configuration was used, which is thought to be more powerful than monophasic stimulation, in particular in producing MEPs [43].

Electromyography (EMG) was performed with silver/silver-chloride disposable self-conductive and self-adhesive surface electrodes. The active electrode was positioned on the belly of the target muscle (FDI), the reference at the metacarpal–phalangeal joint of the index finger, whereas the ground on the dorsal surface of the wrist. For the conduction study of motor nerves, i.e., compound motor action potential (CMAP) and F-waves of the ulnar nerve, a bipolar nerve stimulation electrode, with an interelectrode separation of 25 mm and 6-mm diameter felt pads, was used while recording from the FDI muscle.

**Fig 1:**
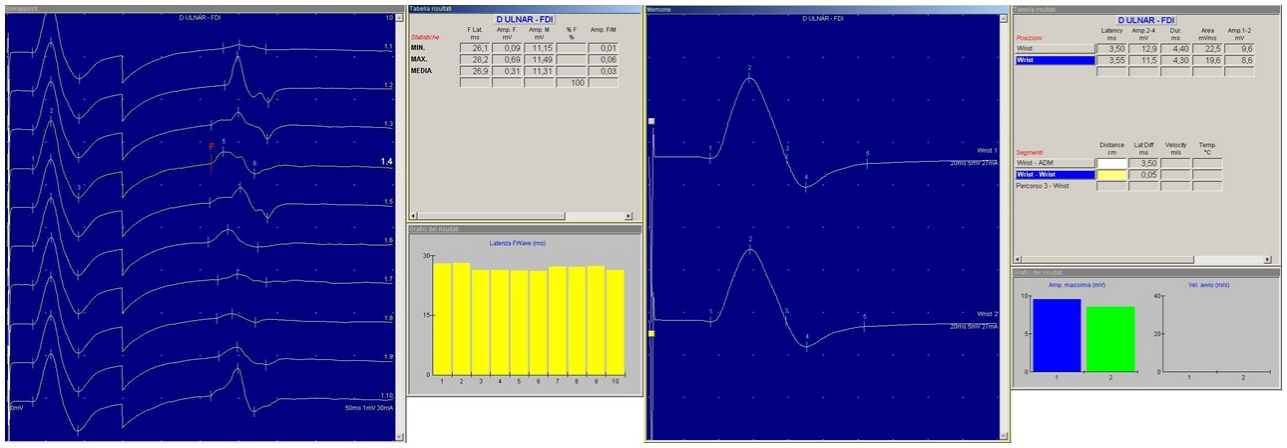
Examples of the compound motor action potential (CMAP) (*right panel*) and F-waves recorded in this study, including the F-wave persistence (*left panel*). (*in alphabetical order*): Amp. F = F-wave amplitude; Amp. F/M = amplitude ratio between F-wave and M-wave amplitudes; Amp. M = M-wave amplitude; Amp. massima = maximum amplitude; D ULNAR FDI = ulnar nerve recorded from the right first dorsal interosseous muscle; Dur. = duration; F Lat. = F-wave latency; Latenza = latency; MAX. = maximum value; MEDIA = mean value; MIN. = minimum value; % F = F-wave persistence.

The resting motor threshold (rMT) was defined as the minimum intensity of stimulation capable to induce, at rest, a MEP amplitude >50 μV in 5 of 10 trials, as recommended by the IFCN guidelines [44]. The central motor conduction time (CMCT) was estimated by subtracting the time of conduction along the peripheral nerve, calculated with the F-wave technique, from the MEP latency recorded during moderate muscular contraction, with an intensity of stimulation of 130% with respect of the rMT. F-waves and peripheral CMAP were evoked with electrical supramaximal stimulations of the right ulnar nerve at wrist. The MEP size was measured as a percentage of supramaximal CMAP size (i.e., the amplitude ratio), which provides a more reliable estimation than the peak-to-peak MEP size [44]. Supramaximal CMAP also refers to the maximum massed action potential (M-wave), which is the electrical equivalent of the recruitment of all motor units within the motor neuron pool [45]. Fig 1 shows examples of the CMAP and F-waves recorded in this study, including the F-wave persistence.

SAI was studied using the technique described by Tokimura and colleagues [26]. For this purpose, a high-voltage Digitimer Stimulator, model DS7A (Digitimer Ltd, Welwyn Garden City, UK), was used. Conditioning peripheral stimuli consisted of single pulses of electrical stimulation (200 μs duration) applied through bipolar electrodes to the median nerve at the wrist (cathode proximal). Paired stimulation was obtained with a 70-mm figure-of-eight coil through a BiStim module (The Magstim Company, Whitland, Dyfed) connected to a CED Micro 1401 interface (Cambridge Electronic Design, Cambridge, UK) allowing stimulus generation and data capture.

**Fig 2:**
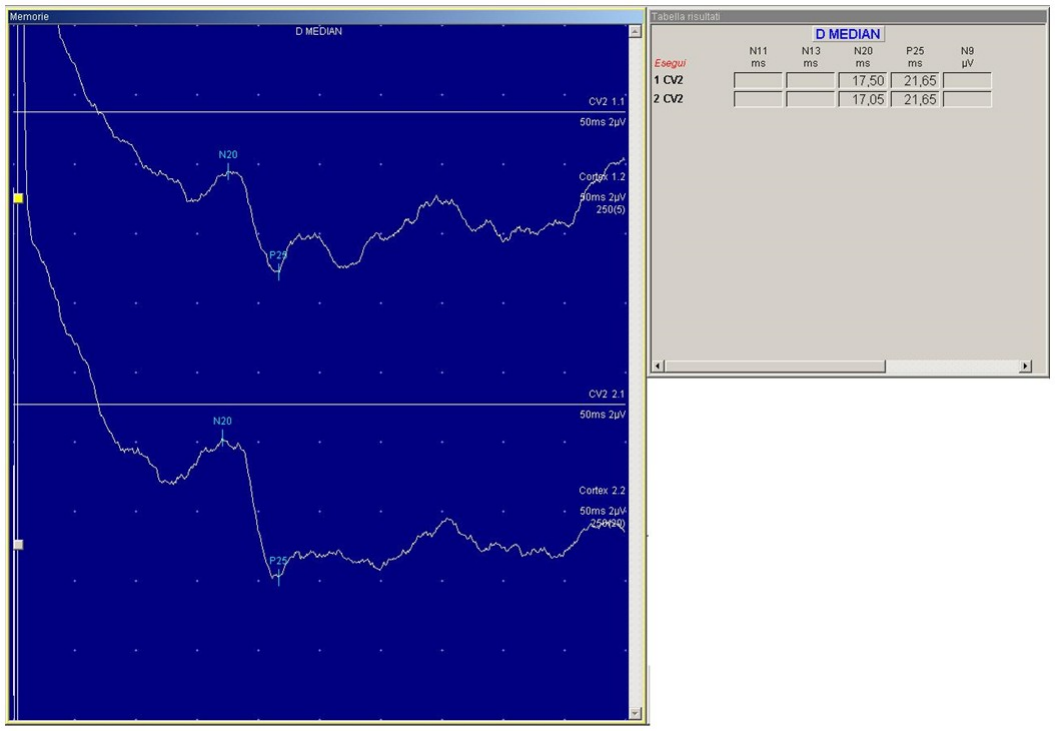
Example of the traces of the averaged SEP recorded in the present study. D MEDIAN = right median nerve.

According to the IFCN guidelines [44], the intensity of the conditioning peripheral nerve stimulation was set just above the motor threshold necessary to evoke a visible twitch of the thenar muscles. The afferent inhibition induced by the peripheral conditioning stimulus was tested at different ISIs. ISIs were based on the latency of the N20 component of the somatosensory evoked potentials (SEPs) obtained from the left hemisphere after stimulation of the median nerve of the dominant hand. To record SEPs, the active electrode was placed 3 cm posterior to C3 (according to the 10–20 International EEG system) and the reference electrode on the forehead. A total of five hundred responses were obtained and averaged from two different trials (250 each) to identify the optimal latency of the N20 peak. An example of the averaged SEP recorded is shown in Fig 2.

ISIs at the latency of N20 plus 2 ms (N20+2) and N20 plus 8 ms (N20+8) were investigated, given that at these intervals it is known to occur a clear inhibition of MEPs and cortical–spinal volleys evoked by TMS [26, 46]. Ten repeats were delivered for cortical stimulation alone, without electrical peripheral stimulation (ISI = 0 ms; unconditioned MEP) and for conditioned stimulation at each ISI, in a pseudorandomized order and with an inter-trial interval of 10 s, as recommended [44]. An algorithm, whose properties approximate the sequence of random numbers (pseudorandom number generator), was used [47]. SAI was measured as the amplitude ratio between conditioned (N20+2; N20+8) and unconditioned MEP response, expressed in percentage (N20+2 ratio, %; N20+8 ratio, %).

A standardized safety checklist was used to screen all individuals before TMS execution [39] and to exclude any condition or medication possibly affecting the CNS excitation state. All procedures were performed with participants seated in a dedicated armchair with constant EMG monitoring to guarantee a desirable level of tonic EMG activity during contraction or a total muscle relaxation. Once collected, data were stored on a dedicated PC by means of an *ad hoc* software that allows to acquire, process, and analyze data [48]. To reduce the interindividual variability, TMS recordings were performed in the same lab and experimental conditions, at the same time of the day (~ 11:30 a.m.), and by the same trained operators (G.L. and F.F.).

### Statistical analysis

Given the non-normal distribution of most data, non-parametric statistics were adopted. The Mann–Whitney test for independent datasets was used for between group comparisons. In order to avoid missing significant differences due to the relatively low number of individuals recruited, we also calculated the effect size of all differences between patients and controls with the rank-biserial correlation by Wendt (*r* = 1−(2U)/(n1 n2)) [49]. With this approach, an *r* of 0.1 is considered as “small”, 0.3 “medium”, and 0.5 “large”. The Freedman ANOVA for within group comparisons was also used and frequencies were analyzed by means of the chi-square test. A *p* <0.05 was set as a statistically significant value.

## Results

Table 1 summarizes all clinical and serological features, as well as data from the main diagnostic exams. The right-handedness of all participants was confirmed by the EHI. General examinations were basically unremarkable in all participants. Apart from a patient with isolated symmetric brisk tendon reflexes at the upper limbs (without any pathological reflex), neurological exams were all normal. Three CD patients had comorbidities: autoimmune thyroiditis (one), Raynaud phenomenon (one), and fibromyalgia and psoriasis (one). All subjects were drug-free, except for one patient taking L-thyroxine, with normal levels of thyroid hormones. Brain CT ruled out intracranial calcifications and clear radiological abnormalities in all patients. As shown in Table 2, the two groups were comparable for age, sex, anthropometric features (height, weight, and body mass index), and educational level. Scores at MoCA and HDRS were significantly worse in patients than in controls.

**Table 1:** Main clinical, laboratory, and instrumental findings in patients with celiac disease.

No.	Age, years	Sex	Family history	Disease duration (years)	Clinical features	Comorbidities	Antibodies	Endoscopy	Histopathology
1	55	F	+	3.5	Tiredness, dyspepsia, weight loss, iron deficiency anemia	-	tTG, EMA	Scalloped duodenal folds	3c
2	18	F	+	1.5	Asthenia, iron deficiency anemia	-	tTG, EMA	Scalloped duodenal folds	3c
3	25	F	+	5.5	Tiredness, iron deficiency anemia, dermatological manifestations	-	tTG, EMA	Scalloped duodenal folds	3c
4	18	F	-	5.0	Headache, tiredness, belly pain, iron deficiency anemia	-	tTG, EMA	Scalloped duodenal folds	3c
5	29	M	+	- (familial screening)	- (familial screening)	-	tTG, EMA	Scalloped duodenal folds	3c
6	45	M	-	3.5	Tiredness, weight loss, headache, iron deficiency anemia, abdominal pain	-	tTG	Scalloped duodenal folds	3c
7	36	F	-	1.5	Headache, tiredness, iron deficiency anemia, vitamin D deficiency weight loss	Autoimmune thyroiditis	tTG, EMA	Scalloped duodenal folds	3c
8	27	F	-	6.0	Abdominal pain, diarrhea, tiredness, unsteadiness, weight loss, iron deficiency anemia	-	tTG, EMA	Scalloped duodenal folds	3c
9	35	F	-	3.5	Abdominal pain, diarrhea, nausea, iron deficiency anemia, tiredness	-	tTG, EMA	Scalloped duodenal folds	3c
10	44	F	+	6.0	Iron deficiency anemia, stypsis and diarrhea, headache, tiredness	Fibromyalgia, psoriasis	tTG	Scalloped duodenal folds	3c
11	45	F	-	1.5	Diarrhea, abdominal discomfort, tiredness	Raynaud phenomenon	tTG	Moderate atrophic villi	3b
12	41	F	-	1.0	Dyspepsia, iron-deficiency anemia, diarrhea, weight loss, tiredness, diffuse pain	-	tTG, EMA	Scalloped duodenal folds	3c
13	49	F	-	5.5	Alternate alvus, dyspepsia, asthenia, tiredness	-	tTG	Scalloped duodenal folds	3c
14	24	F	-	4.0	Tiredness, dyspepsia, weight loss, iron deficiency anemia	-	tTG, EMA	Scalloped duodenal folds	3c
15	20	F	-	3.0	Tiredness, iron deficiency anemia	-	tTG, EMA	Scalloped duodenal folds	3c

EMA = endomysial antibodies; F = female; M = male; tTG = tissue transglutaminase antibodies; Classification of histopathology according to the Marsh–Oberhuber grading system: 3a = mild villous flattening; 3b = severe villous flattening; 3c = complete villous flattening; + = positive/present; − = negative/absent.

**Table 2:** Comparison of demographic features and TMS data of both patients and controls.

Variable	Healthy controls		Celiac disease		Mann-Whitney	Effect size
	(n = 15)		(n = 15)			
	*Mean*	*SD*	*Mean*	*SD*	*p*	*Wendt’s r*
Age, years	33.80	9.29	34.07	12.03	0.852	-0.025
Height, cm	1.68	0.09	1.62	0.08	0.075	0.613
Weight, Kg	60.07	8.19	57.87	17.38	0.135	0.162
BMI, Kg/m^2^	21.32	2.24	21.85	5.99	0.384	-0.116
Education, years	15.87	4.44	14.60	3.44	0.106	0.319
MoCA	28.00	1.00	25.80	2.40	**0.0062**	1.198
HDRS	2.87	2.20	8.27	6.30	**0.0079**	-1.145
rMT, %	36.80	6.41	37.13	5.58	0.917	-0.055
MEP latency, ms	20.33	1.56	19.96	1.24	0.575	0.265
CMCT, ms	6.43	0.89	6.15	0.85	0.395	0.314
MEP amplitude, mV	5.50	1.79	4.47	1.22	0.089	0.674
CMAP amplitude, mV	21.34	6.59	19.84	4.19	0.724	0.272
CMAP distal latency, ms	3.90	0.76	3.41	0.37	0.071	0.828
Amplitude ratio (MEP/CMAP)	0.27	0.11	0.24	0.09	0.184	0.357
F-wave latency, ms	27.79	2.83	27.05	2.07	0.787	0.299
F-wave amplitude, mV	0.13	0.06	0.10	0.04	0.245	0.464
CMCT-F, ms	4.99	0.89	5.23	1.01	0.576	-0.256
N20 latency, ms	18.87	1.36	18.53	1.46	0.547	0.237
Unconditioned MEP amplitude, mV	1.84	0.71	1.53	0.68	0.281	0.438
N20+2 amplitude, mV	1.04	0.64	0.99	0.57	0.884	0.080
N20+8 amplitude, mV	1.75	1.00	1.38	0.68	0.431	0.421
N20+2 ratio, %	56.03	27.09	70.45	37.82	0.340	-0.438
N20+8 ratio, %	95.35	33.11	99.17	55.36	0.431	-0.084

BMI = body mass index; CMAP = compound motor action potential; CMCT = central motor conduction time; CMCT-F = central motor conduction time estimated by means of the F-waves; HDRS = 17-item Hamilton Depression Rating Scale; SD = standard deviation; MEP = motor evoked potential; MoCA = Montreal Cognitive Assessment; N20 = cortical component of the somatosensory evoked potential obtained after stimulation of the median nerve of the dominant hand; NS = not significant; rMT = resting motor threshold; N20+2/+8 = short-latency afferent inhibition at the interstimuls interval of 2 and 8 ms, respectively; N20+2/+8 ratio, % = amplitude ratio between the conditioned and the unconditioned MEP response, expressed in percentage, at the interstimuls interval of 2 and 8 ms, respectively; TMS = transcranial magnetic stimulation; bold numbers = statistically significant *p* values.

Table 2 also shows the comparison of TMS data between the two groups; no statistically significant difference was observed for all measures, including SAI at both ISIs. Namely, among CD patients, 9 exhibited inhibition of the MEP responses (amplitude ratio between conditioned and unconditioned MEP <1) and 6 facilitation (amplitude ratio between conditioned and unconditioned MEP >1) at ISI 2 ms, whereas 7 patients inhibited and 8 facilitated at ISI 8 ms (chi-square = 0.14, *p* = 0.712). Among healthy controls, 7 exhibited inhibition and 8 facilitation of the MEP responses at ISI 2 ms, whereas 4 patients inhibited and 11 facilitated at ISI 8 ms (chi-square = 1.29, *p* = 0.256). Within-group ANOVA comparison between unconditioned and conditioned MEP responses for the two groups resulted to be significant for both patients (chi-square = 13.733, *p* = 0.001) and controls (chi-square = 16.533, *p* = 0.00026), thus confirming the significant variation of SAI in each group.

## Discussion

### Main findings

This is the first study that explores SAI to TMS in newly diagnosed CD patients. The main finding is that the central cholinergic functioning, as explored by the SAI of the motor cortex, does not seem to be affected in this *de novo* CD sample with respect to age-matched healthy controls. Both groups, indeed, exhibited a comparable level of SAI at two different ISIs, a finding that paralleled the lack of a clear cognitive impairment. Coherently, although MoCA scored significantly worse in patients, the mean value was still within normal limits. Overall, the pathomechanisms underlying these results seem to be rather complex. To date, indeed, no study based on TMS or other techniques has shown any involvement of the central acetylcholine or cholinergic fibers within the CNS in CD.

Only few investigations have applied MEPs in CD to specifically evaluate the TMS profile of cortical excitability. In the first study on 20 *de novo* patients and 20 age-matched controls [50], we showed a hyperfacilitation and a disinhibition of the M1 in patients, suggesting an impaired glutamatergic and GABAergic circuitry, respectively. Unbalanced inhibitory and excitatory transmissions within the M1 was hypothesized to correlate with a cross-interaction between some neuronal antigens and gliadin antibodies. Similarly, the CNS-produced antibodies against glutamic acid decarboxylase may have impaired GABAergic interneurons activity [50]. The same sample was re-assessed after a relatively short-term GFD (median 16 months) [51]. Gastrointestinal manifestations improved but, unexpectedly, the excitation state of M1 to TMS enhanced further. This result was thought to be an index of an adaptive re-modeling of the motor areas, probably not related to the GFD. It was also hypothesized that the duration of the diet, or its adherence, was not optimal to produce a significant recovery [51]. In a further study following a considerably longer gluten restriction (mean period 8.35 years), we revealed that only a sustained GFD could restore the TMS-associated modifications in adults with CD. However, some excitatory changes persisted, likely indicating a synaptic intracortical rearrangement of the “celiac brain”, mostly involving the glutamate-mediated interneurons [52]. Recently, the interhemispheric excitability by the transcallosal inhibition was evaluated, as reflected by the duration and latency of the ipsilateral silent period (iSP) to TMS, in a sample of newly diagnosed CD patients [53]. We found that iSP was significantly shorter in patients than controls, with a positive correlation between MoCA score and iSP duration, suggesting an interhemispheric motor disinhibition and supporting the involvement of GABA [53]. Taken together, these results support the interplay between GABAergic and glutamatergic circuits previously explored by using the paired-pulse TMS paradigm [54].

In the sample studied here, although the statistically significant difference in MoCA score, a clear cognitive impairment was not clinically evident in CD patients. However, an impairment of SAI in the more advanced stages of CD, especially if the GFD is not promptly adopted, cannot be excluded. Of note, although the pathogenesis of cognitive difficulties in CD remains unclear, it has been shown that pathomechanisms may be more likely related to the model of vascular dementia (VaD) than to the dysfunction of cholinergic pathways, as classically seen in AD [55]. In particular, the so-called “gluten encephalopathy” seems to be mediated by a vascular inflammation with an aetiology different from that of the cholinergic dysfunction [55]. This might be a relevant factor to consider for explaining the lack of difference we observed between the two groups regarding SAI, which is known to be reduced in AD (including the early stage) and in amnestic MCI [56], but not in most patients with VaD [57] or in those mild vascular cognitive impairment [58]. Additionally, the fact that central cholinergic fibers do not seem to be affected at this stage does not prevent from the involvement of other neurotransmission pathways, such as glutamate or GABA, that may be implicated in the occurrence of cognitive and mood disorders in CD patients, as observed by previous studies both before and after GFD [59, 60]. This is also in line with the fact that SAI can be influenced by other neurotransmitter systems (such as GABA) in addition to acetylcholine [31] and this finding should be considered when interpreting the lack of change of SAI observed in patients at this stage.

It is worth to remind that SAI is obtained at relatively short ISIs (20–25 ms), but further manipulating the latency between the two TMS stimuli alters the effect on cortico-spinal excitability. In particular, it is known that progressively increasing ISI results in afferent facilitation (at ISIs of 25–80 ms) and eventually (at ISIs of 100–1,000 ms) in long-latency afferent inhibition (LAI) [26, 61, 62]. The magnitude of SAI and LAI is directly related to the amplitude of the sensory afferent volley, such that greater inhibition is observed as a larger volume of sensory afferents are recruited [63, 64], thus likely reflecting cortical rather than spinal inhibitory mechanisms [26, 61, 65]. These TMS measures are frequently referred to as markers of sensory-motor integration due to their nature of acquisition, such that ascending sensory input is integrated within the sensory-motor cortex to alter descending motor output [66]. However, unlike SAI, it is currently unknown whether LAI could be also modulated by acetylcholine [66]. Moreover, although both measures have shown moderate intersession reliability [67], LAI exhibited larger measurement error than SAI [68]. As such, to sufficiently reduce this error, LAI assessment may require a sample size larger than that of the present study.

Regarding psychiatric comorbidities, anxiety and depression in particular, are frequently associated with CD [69, 70]. In our patients, depressive symptoms were higher than in controls, although the mean HDRS score was suggestive of a mild depression. Nevertheless, depressive disturbances can substantially affect the quality of life of CD patients and are a marker of poor adherence to the diet [71]. Screening and following up for depressive symptoms are therefore crucial to promptly suggest appropriate pharmacotherapy and/or psychological support. Of note, accumulating evidence has suggested that some inflammatory soluble factors derived from the inflamed intestinal mucosa across the gut–epithelial barrier and the blood–brain barrier are major factors for structural and functional alterations in the CNS. In particular, neuroinflammation has been shown to exert detrimental effects on both cognition and emotional behavior [72, 73], thus supporting the link between neuroinflammatory, neurological, and psychiatric manifestations in CD. Translationally, these findings highlight the importance of a prompt diagnosis, clinical awareness, and compliance to an adherent GFD to prevent, or at least limit, the neurological and neuropsychiatric involvement in CD and the related disease progression [7].

Finally, although no specific neurochemical study has been performed in CD, some metabolic changes seem to be present in these patients. Metabolomics studies, despite being very limited and restricted to the use of nuclear magnetic resonance-based methods, suggest minor but significant alterations in energy metabolism, lipid metabolism, and microbiome-derived metabolites [74–80]. In particular, different concentrations of methionine, choline, and choline-derived lipids in CD are described [74–76, 78, 79] and, as known, choline can also be acetylated into acetylcholine by the choline acetyltransferase enzyme. However, targeted metabolomic analysis investigating differences in the plasma choline/methionine metabolome of CD subjects were not reported. Only one study [81] in 17 children with CD, treated with a GFD, and 17 healthy control siblings has recently demonstrated the persistence of defects in the trans-sulfuration pathway of CD, despite dietary treatment. Conversely, choline metabolism seem to be preserved [81], a finding that might add further support to the results of the present study.

### Limitations

The main limitation, as usually occurs in TMS research, is the relatively small sample size, although the patients were carefully screened and selected, they were homogenous for clinical-serological features and histopathological findings, were all *de novo* and drug-free, and matched for age, sex, and education with healthy subjects. Nevertheless, since SAI has been shown to decrease with age [82], the age range of the participants enrolled here seems to be large. Therefore, it cannot be excluded that a difference between CD and controls would have appeared if a group of older participants was tested. As such, this aspect deserves further replication in future studies.

Another caveat is that, since TMS provides a functional evaluation of the cortical activity but not of structural changes, a detailed morphological assessment of the cerebral cortex and the cortico-spinal tract were not performed, thus precluding correlations with neuroimaging data. Although we have excluded clear neuroimaging abnormalities in all patients, brain CT remains a gross radiological exam, able to properly detect intracranial calcifications better than MRI, but with general low sensitivity and specificity. The same holds true for an extensive neuropsychological battery of tests, which was limited to an only but comprehensive screening tool (MoCA).

Third, based on the cross-sectional design of the study, it should be acknowledged that a causal relationship between SAI and CD cannot be established beside, at this stage, an association only between specific TMS parameters and gluten exposure.

Finally, although SAI is considered to be a rather reliable measure, there is ultimately a degree of uncertainty on what is being precisely reflected when a difference is found or not found (as in the present study). This feature, which is shared by other TMS indexes and, more in general, by most of the neurophysiological tests, should be taken into account when interpreting these findings.

## Conclusions

Central cholinergic pathways to TMS do not seem to be functionally involved in *de novo* CD patients, who do not show an overt cognitive impairment or depressive disorder. Longitudinal studies correlating clinical, TMS, and neuroimaging data, both before and after GFD, are needed.

## Abbreviations

AD: Alzheimer’s disease
CD: celiac disease
CMAP: compound motor action potential
CMCT: central motor conduction time
CNS: central nervous system
CT: computed tomography
EHI: Edinburgh Handedness Inventory
EMG: electromyography
FDI: First Dorsal Interosseous
GABA: gamma-aminobutyric acid
GFD: gluten-free diet
HDRS: 17-item Hamilton Depression Rating Scale
IFCN: International Federation of Clinical Neurophysiology
ISI: interstimulus interval
iSP: ipsilateral silent period
LAI: long-latency afferent inhibition
M1: primary motor cortex
MCI: mild cognitive impairment
MEP: motor evoked potential
MoCA: Montreal Cognitive Assessment
MRI: magnetic resonance imaging
rMT: resting motor threshold
SAI: short-latency afferent inhibition
SD: standard deviation
SEP: somatosensory evoked potentials
TMS: transcranial magnetic stimulation
VaD: vascular dementia
